# No drains in thoracic surgery with ERAS program

**DOI:** 10.1186/s13019-020-01164-5

**Published:** 2020-05-24

**Authors:** Cheng Shen, Guowei Che

**Affiliations:** Department of Thoracic Surgery, West-China Hospital, Sichuan University, Chengdu, 610041 China

**Keywords:** Enhanced recovery after surgery, Bladder catheterization, Chest tube

## Abstract

Enhanced recovery after lobectomy surgery (ERAS) concept has been greatly developed between clinical implementation and minimally invasive surgery. In addition to the minimally invasive surgery, the management of the perioperative catheter has also attracted everyone’s attention. Tubeless minimally invasive treatment includes no urinary catheter placement during the operation and no chest tube after the operation. Here, we summarized all the reports on no urinary catheterization and no chest tube in patients with thoracic surgery and the impact of postoperative length of stay (LOS) and postoperative complications. We find that avoiding chest drain and urinary catheter placement after the surgery appears to be safe and beneficial for patients.

## Background

VATS is currently the common method of lobectomy or wedge resection for thoracic surgeons. At the same time, minimally invasive surgery is one of the main means of enhanced recovery after surgery (ERAS) [1–3]. Through the cooperation with multi-disciplinary departments, the ERAS concept has been greatly developed between clinical implementation and minimally invasive surgery. Tubeless minimally invasive treatment includes no urinary catheter placement during the operation and no chest tube after the operation. Here, we summarized all the reports on no urinary catheterization and no chest tube in patients with thoracic surgery and the impact of postoperative length of stay (LOS) and postoperative complications.

## Main text

In addition to the minimally invasive surgery, the management of the perioperative catheter has also attracted everyone’s attention [4]. Traditionally, bladder catheterization and chest tube are a routine procedure in treatment after general anesthesia in patients with thoracic surgery. If the patient removes the bladder catheterization early after the operation, it may lead to postoperative anesthesia dysuria. It is recommended to indwell the catheter after surgery for several days. However, the clinical drawbacks of this method are also obvious: Firstly, the patient’s comfortability is significantly reduced, especially the Catheter-Related Bladder Discomfort (CRBD) in patients undergoing a urinary catheter insertion intraoperatively; secondly, the patient’s activity is limited, which is not conducive to leave bed early and rapid recovery; thirdly, it will increase the occurrence of postoperative urinary retention (POUR) [5]. Recently, some clinically relevant studies have shown that performing a certain fluid management during surgery can control the amount of urine, and inserting a catheter to monitor perioperative urine volume may not be an essential operation during the perioperative period. No urinary catheter placement during the operation is one of the core elements of tubeless minimally invasive treatment [4, 6].

The placement of chest tube after surgery will increase the feeling of chest pain and rise the amount of analgesic used in patient, even extend the length of hospital stay (LOS) [4]. More importantly, it will affect the early activity of patient after surgery. Although it has been reported that surgeons attempt to shorten the time of postoperative chest tube drainage by controlling the occurrence of postoperative complications, the guidelines for the operation of thoracic surgery do not suggest omitting the placement of chest tube after the surgery [7]. Combined with a small number of published studies, early chest extraction or no chest tube have better perioperative outcomes in patients compared with conventional 28F chest tube placement. Based on cases of non-intubated VATS group comparing to general chest tube group, Cui et al [8] found that a significantly decreased postoperative pleural drainage volume could be expected in tubeless patients and they found significantly decreased inflammatory cytokine levels in the lung and decreased lung infection in comparison to the results for the bronchoalveolar lavage fluid and serum inflammatory cytokines. All these modalities can make thoracic day surgery a reality.

So we searched all of the articles that were published from October 2000 to February 2020 in the PubMed, Web of Science, EMBASE and CNKI databases and only several researches were involved in the present study. Here, we summarized all the reports on no urinary catheterization and no chest tube in patients with thoracic surgery and the impact of postoperative length of stay (LOS) and postoperative complications (Tables 1 and 2). The guideline of Newcastle-Ottawa Scale (NOS) was used for evaluating this research including three perspectives of selection, comparability and exposure. The assessment tool including the star system, a maximum of 9 stars, was used in this research. Specific evaluation system is that 8–9 stars are high quality; 6–7 stars are reasonable quality, and 6 stars or less are bad.

**Table 1:** Summary of articles reporting on no urinary catheterization in patients with thoracic surgery

Author	YOP	Study Period	Study Type	TP	NUCP	UCP	NUC Group				UC Group			
							OT (min)	P-LOS (day)	UR	UTI	OT (min)	P-LOS (day)	UR	UTI
Lai [5]	2019	2014–2017	Retrospective: cohort study	2495	660	1835	115	4	74 (11.2%)	38 (5.7%)	120	5	136 (7.4%)	153 (8.3%)
Peng [6]	2017	2014–2015	Retrospective: case series	43	40	–	22 ± 5	–	0 (0%)	0 (0%)	–	–	–	–
Li [4]	2017	2012–2014	Retrospective: case series	34	34	–	42 ± 10	1 ± 1	0 (0%)	0 (0%)	–	–	–	–
Yang [9]	2016	2015–2016	Retrospective: cohort study	148	74	74	–	4.4 ± 1.0	–	–	–	6.1 ± 2.0	–	–
Qiu [10]	2016	2015–2016	Retrospective: cohort study	148	74	74	96.7 ± 30.4	4.0 ± 1.1	5 (6.7%)	1 (1.3%)	107.2 ± 28.4	6.2 ± 1.0	7 (9.4%)	3 (4.0%)
Xu [11]	2016	2014–2015	Prospective cohort study	133	65	68	–	5.0 ± 1.6	3 (4.6%)	6 (9.2%)	–	6.5 ± 3.1	7 (10.2%)	18 (26.4%)
Qiu [12]	2015	2014.4–2014.12	Prospective cohort study	100	50	50	–	5.0 ± 1.5	2 (4.0%)	6 (12.0%)	**–**	**–**	5 (10.0%)	18 (36.0%)

*YOP* year of publication, *TP* total patients, *NUCP* non-urinary catheter patients, *UCP* urinary catheter patients, *OT* operative time, *P-LOS* postoperative length of stay, *UR* urinary retention, *UTI* urinary tract infection

**Table 2:** Summary of articles reporting on no chest tube in patients with thoracic surgery

Author	YOP	Study Period	TP	NCT	CT	NCT Group							
						OT	BL (mL)	OM	LOS	PN	SE	PT	AT
Cheng [13]	2019	2014–2018	282	246	36	48 ± 6	3 ± 0.4	VATS	2	–	202 (82.11%)	2 (0.8%)	–
Watanabe [14]	2017	1998–2002	93	42	34	–	–	VATS WR	3.2 ± 1.0	–	–	2 (4.7%)	–
Murakami [15]	2017	2012–2014	162	102	60	–	–	VATS	9.7 ± 3.8	3 (1.8%)	–	–	5 (3.0%)
Steunenberg [16]	2017	2011–2014	49	28	21	–	–	VATS WR	3	1 (3.5%)	–	2 (7.1%)	–
Lu [17]	2016	2013–2015	89	44	45	–	–	VATS WR	3.1 ± 0.9	–	15 (16.8%)	0 (0%)	–
Yang [7]	2016	2015–2016	60	30	60	72 ± 21	–	Uniportal VATS WR	3.1 ± 0.7	–	2 (6.6%)	–	–
Holbek [18]	2016	2015	166	51	0	36	–	VATS WR	1	–	2 (1.2%)	–	–
Cui [8]	2016	2012–2016	173	21	19	37 ± 11	20.8 ± 15.7	VATS	1.5 ± 0.7	–	–	2 (9.5%)	–
Ueda [19]	2013	2011–2012	50	29	21	152 ± 53	63 ± 62	VATS	–	–	–	–	–
Nakashima [20]	2010	2000–2009	333	132	201	–	–	VATS WR	4.6 ± 2.2	–	–	10 (7.5%)	–

*YOP* year of publication, *TP* Total Patients, *NCT* No chest tube, *CT* Chest tube, *M* male, *F* female, - Not report, *VATS*, video-assisted thoracic surgery, *LOS* length of stay, *PN* Pneumonia, *SE* Subcutaneous emphysema, *PT* Pneumothorax, *AT* Arrhythmia, *OT* Operation time, *WR* wedge resection, *OM* Operation method, *BL* Blood loss

The key point of ERAS emphasizes the optimization management of perioperative procedures. The clinical focus is on optimizing patient care processes such as shortening the time of examination and tubeless. The reducing postoperative complications and shortening LOS as a criterion for evaluating the feasibility of the ERAS program by most of clinical surgeons [21].

As seen in our Table 1, two researchers mentioned the relationship between operation time and urinary catheterization. Lai et al [5] and Qiu et al [10] reported that there was no significant statistical difference between indwelling catheter and operation time. In the time of postoperative LOS, it showed that there was a statistically significant difference between the NUC group and the UC group, especially in the Li *et al’s* report [4], 34 patients with small pulmonary nodules were treated using VATS and no urinary catheter during the operation with only one postoperative day. For patients undergoing VATS thoracic surgery, no urinary catheter during the operation was associated with significantly shorter hospital stay.

POUR is clinically defined as acute urinary retention is one of the common complications after the surgery. To develop an enhanced recovery pathway to improve efficiency, shortening LOS is important in terms of quality of patient care, but also healthcare costs. The diagnosis of POUR has clinical implications such as delayed discharge, prolonged LOS, potential risk of systemic infection from urinary catheterization and possible long-term bladder dysfunction [5]. In addition, the results of the 2019 study showed that the incidence of UTI in NUC group was significantly lower than in UC group. In the study, the authors further analyzed the risk factors of UTI and found that indwelling catheter was an independent risk factor for UTI. This result suggests that the insertion of urinary catheter can reduce the occurrence of UTI. It may also be one of the important reasons for not performing catheterization during thoracic surgery.

As seen in our Tables 2 and 3, all the patients without chest tube underwent with thoracoscopic minimally invasive surgery and over half of the operation method is VATS wedge resection. Yang *et al* [7] reported a retrospective review that patients with lung cancer underwent Uniportal VATS without chest tube after the surgery, which reduced postoperative pain, residual paresthesia, and LOS compared with multiportal VATS. NCT with the promotion of minimally invasive surgery, especially in VATS pulmonary wedge resection is safe for selected patients, compared with traditional conventional thoracotomy, postoperative complications are relatively low. However, we should pay more attention to the results of the NCT in VATS lobectomy in the future. In addition, the incidence of postoperative complications in patients undergoing minimally invasive surgery is still 20%, and postoperative patient mortality due to complications reaches 10%. Therefore, finding the influencing factors of postoperative complications and taking appropriate measures is the key to consolidate the results of surgical treatment. The common postoperative complications in pulmonary surgery includes pneumonia, subcutaneous emphysema, pneumothorax and arrhythmia. As reported in Cheng *et al*’s research, there were 202 asymptomatic subcutaneous emphysema patients, and the asymptomatic subcutaneous emphysema spontaneously resolved within 3 to 7 days [13].

**Table 3:** Summary of articles reporting on chest tube in patients with thoracic surgery

Author	YOP	Study Period	TP	NCT	CT	CT Group							
						OT	BL (mL)	OM	LOS	PN	SE	PT	AT
Cheng [13]	2019	2014–2018	282	246	36	48 ± 6	3 ± 0.4	VATS	2	–	202 (82.11%)	2 (0.8%)	–
Watanabe [14]	2017	1998–2002	93	42	34	–	–	VATS WR	3.6 ± 1.5	1 (2.9%)	–	1 (2.9%)	–
Murakami [15]	2017	2012–2014	162	102	60	–	–	VATS	12.9 ± 7.8	5 (8.3%)	–	–	4 (6.7%)
Steunenberg [16]	2017	2011–2014	49	28	21	–	–	VATS WR	4	1 (4.7%)	–	1 (4.7%)	–
Lu [17]	2016	2013–2015	89	44	45	–	–	VATS WR	4.1 ± 0.8	–	24 (53.3%)	0 (0%)	–
Yang [7]	2016	2015–2016	60	30	30	79 ± 32	–	Uniportal VATS WR	4.4 ± 1.3	–	–	4 (13.3%)	–
Holbek [18]	2016	2015	166	51	0	–	–	–	–	–	–	–	–
Cui [8]	2016	2012–2016	173	21	19	39 ± 12	24.7 ± 12.3	VATS	3.9 ± 2.7	2 (10.5%)	–	–	–
Ueda [19]	2013	2011–2012	50	29	21	198 ± 78	189 ± 230	VATS	–	–	–	–	1 (4.7%)
Nakashima [20]	2010	2000–2009	333	132	201	–	–	VATS WR	6.7 ± 4.4	–	–	8 (4.0%)	–

*YOP* year of publication, *TP* Total Patients, *NCT* No chest tube, *CT* Chest tube, *M* male, *F* female, - Not report, *VATS* video-assisted thoracic surgery, *LOS* length of stay, *PN* Pneumonia, *SE* Subcutaneous emphysema, *PT* Pneumothorax, *AT* Arrhythmia, *OT* Operation time, *WR* wedge resection, *OM* Operation method, *BL* Blood loss

Hospital hospitalization rates have been shown to be the most important direct cost predictor of patient, which accounts for 31–68% of hospitalization expenses [10, 22]. In Yang *et al*’s report [9], they analyzed the material costs, care costs and specific time associated with indwelling catheters in the NUC and UC groups in detail (including time to place the catheter, time to replace the drainage device, time to observe and care the patient and time to remove the catheter). Material cost for patients in UC group (4811.48 yuan vs. 296.74 yuan, *P* = 0.045), nursing expenses (7413.32 yuan vs. 457.32 yuan, *P* = 0.013) and total cost (12,224.8 yuan vs. 754.06 yuan, *P* = 0.000) were higher than the NUC group. The total period of care in the UC group was longer than that in the NUC group. Tubeless minimally invasive treatment and ERAS for patients saves the time of urethral catheter placement and the daily care of the postoperative urinary catheterization, which significantly reduces the nurses’ workload [23].

## Conclusions

The purpose of the ERAS program is not only to shorten the postoperative LOS, but also to promote the safe recovery of patients. We find that avoiding chest drain and urinary catheter placement after the surgery appears to be safe and beneficial for patients. The implementation of perioperative management measures must be conducted under the guidance of evidence-based medicine to benefit patients. Thoracic surgery without indwelling catheter is not only the embodiment and implementation of the ERAS concept, but also an effective process to improve the patient’s perioperative satisfaction and comfort. At the same time, we should pay attention to preoperative evaluation of the patient’s medical history and surgical methods to assess whether patients need to input the catheter or not. It is necessary to conduct multi-center clinical research in a number of hospitals across the country to obtain clinical evidence.

## Data Availability

All data for this study are publicly available and are ready for the public to download at no cost from the official websites of the PubMed, EMBASE, CNKI and the Web of Science. There is no need to have the formal permission to use data for this study.

## Abbreviations

ERAS: Enhanced recovery after surgery
LOS: Length of stay
CRBD: Catheter-Related Bladder Discomfort
POUR: Postoperative urinary retention
VATS: Video-assisted thoracic surgery
